# Microneedling Technique With Topical Vitamin C for Gingival Depigmentation Enhancing Gingival Aesthetics: A Longitudinal Study

**DOI:** 10.7759/cureus.81338

**Published:** 2025-03-28

**Authors:** Sana Mansuri, Tanvi Hirani, Jay T Patel, Shirishkumar Patel, Dipak Chaudhari, Bhavin Patel, Hiren H Patadiya, Mainul Haque, Santosh Kumar

**Affiliations:** 1 Department of Periodontology, Karnavati School of Dentistry, Karnavati University, Gandhinagar, IND; 2 Department of Periodontology and Implantology, Karnavati School of Dentistry, Karnavati University, Gandhinagar, IND; 3 Department of General Dentistry, My Dental Southbridge PLLC, Southbridge, USA; 4 Department of Pharmacology and Therapeutics, National Defence University of Malaysia, Kuala Lumpur, MYS; 5 Department of Research, Karnavati School of Denistry, Karnavati University, Gandhinagar, IND

**Keywords:** ascorbic acid, collagen production, dr. pen ultima a1, healing, melanin, microchannels, minimally invasive, needle tip, safe, skin rejuvenation

## Abstract

Introduction

As the focus on dental aesthetics intensifies, contemporary cosmetic dentistry seeks to establish an equilibrium between teeth and gums. Gingival hyperpigmentation, resulting from excessive melanin accumulation, can impact individuals regardless of age or gender and frequently gives rise to aesthetic apprehensions. Microneedling combined with topical vitamin C is a promising new method for improving gum appearance, particularly for reducing gingival hyperpigmentation caused by excess melanin. Microneedling is less expensive, more versatile, and promotes faster healing than traditional procedures like lasers or surgery.

Materials and methods

A study with 10 participants at the Karnavati School of Dentistry demonstrated significant pigmentation reduction. A paste was made with powdered ascorbic acid and a liquid ampule solution and applied with a Derma Pen (Dr. Pen Ultima A1, C Cube Advanced Technologies, India), which is a rechargeable pen-like instrument with 24-36 adjustable needles (0.25 mm to 2 mm) and five-speed settings. The gadget enables multi-directional treatment, and its disposable needle tips assure clean use with several patients.

Results

A significant decrease in melanin pigmentation was observed across all age groups, but anatomical challenges, such as irregular gingival contours, hampered results in some cases.

Conclusion

Microneedling is a safe and effective option for improving gingival aesthetics. As the technique evolves, it has the potential for broader applications.

## Introduction

As the need for dental aesthetics advances, today's cosmetic dentistry focuses on addressing structural and physiological problem areas and attaining harmony between the gums and teeth. A beautiful smile relies on teeth' whiteness and gums' pinkness [1]. Healthy gums are usually pink, but their color can vary based on keratinization, gingival thickness, vascularization, and the presence of melanin. Gingival hyperpigmentation is often related to an array of internal and external factors and results in excessive melanin deposition in the epithelial layer [2]. Melanin pigmentation can occur at any stage of life, regardless of sex, and it also determines racial color [3]. Melanin activity, distribution, and pigment degradation all influence the degree of hyperpigmentation [4]. Tyrosinase is an enzyme that converts L-tyrosine to levo-dihydroxyphenylalanine (L-Dopa) and ultimately to dopaquinone present in melanin [1,5]. This kind of darkening of the gingiva is the cause of shame in smile-aware persons, boosting the demand for aesthetic procedures to cure the same [6].

Treatment options for gingival hyperpigmentation can be divided into procedures that remove pigments and other hidden pigments. Pigment removal can be performed by surgical, non-surgical, or chemical methods [7]. These procedures include scalpel surgery, drill wear, laser ablation, cryosurgery, electrocautery, and radiosurgery [8]. All of these treatment options have their possibilities of advantages and disadvantages [8]. Microneedling technology is a non-surgical method known for stimulating collagen production by creating many small punctures in the skin. In recent years, microneedling has become widely used in dermatology due to its effectiveness, simplicity, cost-effectiveness, and therapeutic and cosmetic benefits. The microneedling approach was chosen for the depigmentation procedure because it is less invasive, resulting in smaller lesions than other therapies.

Problem statement of the study

There is limited information on the efficacy of microneedling in conjunction with vitamin C for gingival depigmentation. There is also a shortage of established standards for evaluating pigmentation reduction and healing results. Few ethical studies have looked into the therapeutic potential of this minimally invasive method. Establishing a standardized procedure would help to ensure consistent results and improve the clinical evaluation of gingival aesthetics.

Objectives of the study

This study aimed to determine the relationship between gingival pigment reduction and the microneedling process combined with topical vitamin C and assess its usefulness as a minimally invasive strategy to improve gingival aesthetics.

## Materials and methods

**Figure 1:**
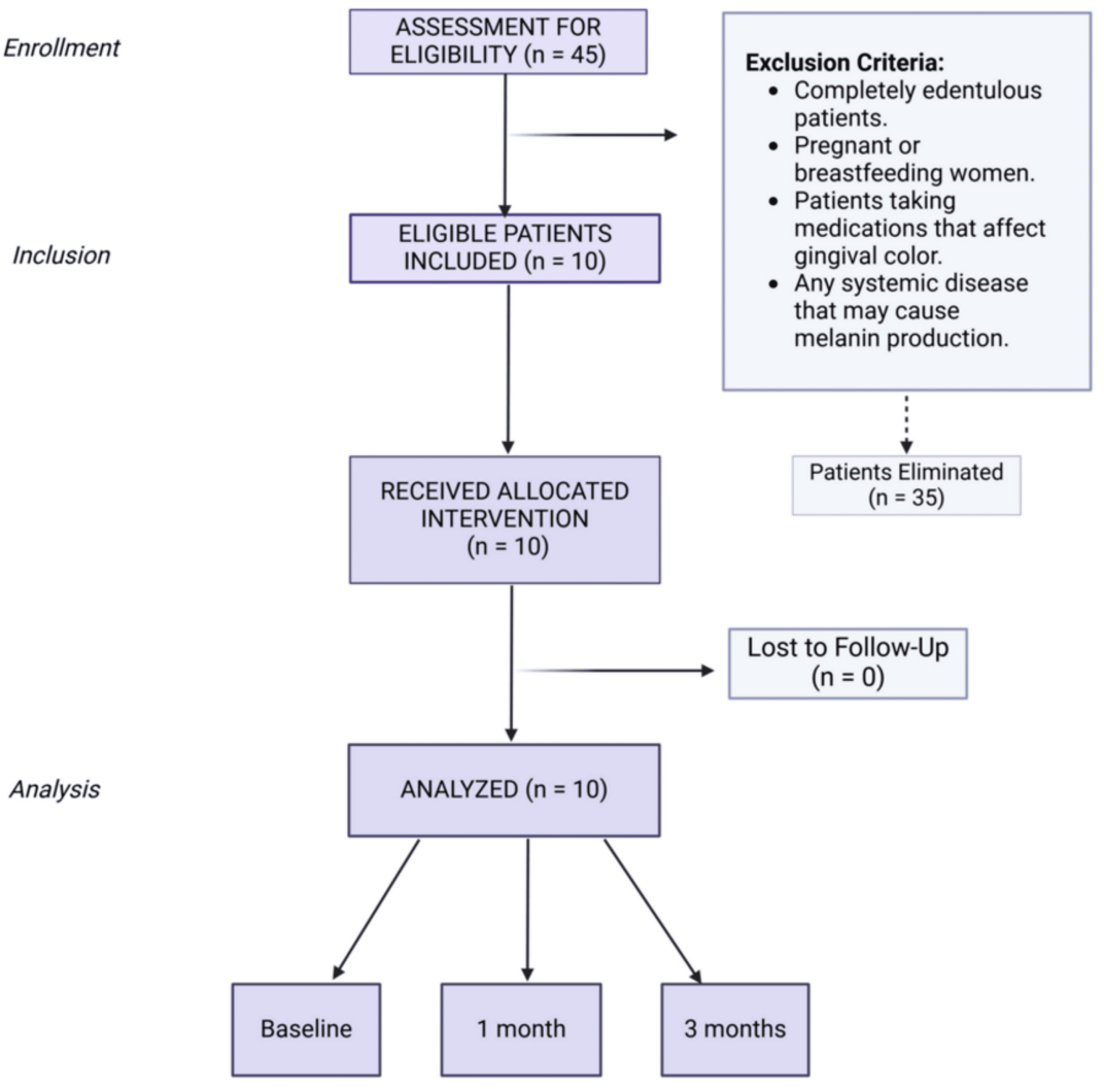
A diagram illustrating the selection process of patients. Notes: This figure was drawn using the premium version of BioRender [9] (https://biorender.com/), accessed on February 19, 2025, with the agreement license number BW27XLLLFF. Illustration Credit: Sana Mansuri.

This longitudinal study was conducted in the Department of Periodontology at Karnavati School of Dentistry, Gujarat, India. The study was conducted basically for aesthetic purposes. The study was started on June 10, 2024 and completed on September 10, 2024. It was evaluated and approved by the institutional review board of Karnavati University, Gandhinagar, Gujarat, India (KSDEC/23-24/Apr/009) on May 6, 2024. The study was conducted per the Declaration of Helsinki of the World Medical Association (2021) guidelines. All patients who agreed to participate in the study got informed written consent. The universal sampling method was adopted. The study included 10 out of 45 patients who fulfilled the requirements for inclusion and exclusion. Ten patients were selected through inclusion and exclusion criteria (Figure 1).

Ten patients who met these criteria were recruited for the current study. The studied participants were between 14 and 30 years old and had received no previous treatments that affected gingival health or color. According to Dummett's classification, the people chosen had mild-to-severe anterior melanin gingival pigmentation and were free of other medical problems. Patients who were entirely edentulous, pregnant or lactating, taking medicines that impact gingival color, and having an underlying medical condition (e.g., systemic diseases (such as, diabetes mellitus, cardiovascular and psychiatric disorders) that hinder the healing process) that causes melanin formation were all excluded. Moreover, edentulous patients were old and sick and did not agree to be study participants. Additionally, aesthetic purpose was not as essential for young study participants.

The study examined parameters - patients were evaluated, and their gingival color was assessed using two indexes. The first index employed was the Dummett-Gupta Oral Pigmentation Index (DOPI) [10], which measured hyperpigmentation intensity on a scale of 0 to 3: 0 for pink gingiva, 1 for mild light brown, 2 for moderate brown or mixed brown and pink, and 3 for intense brown or blue-black. The scoring system for evaluating gingival wound healing includes recording scores on the first and seventh days following surgery. The scale comprises a score of 0, denoting necrotic gingival tissue; a score of 1, indicating ulcers; a score of 2, representing partial or incomplete gingival epithelialization; and a score of 3, signifying complete gingival epithelialization. These scores assess the healing progress after surgical procedures on the gums. We utilized a paste created using powdered ascorbic acid, a liquid ampule solution, and a Dermapen (Dr. Pen Ultima A1, C Cube Advanced Technologies, India), a pen-like device comprising a handle and needles operated by a battery that can be recharged. The needle tip contains 24-36 needles stacked in rows with five-speed options. Although it usually depends on individual cases, we used the same speed in this study. Its handpiece allows the physician to treat regions in any direction; needle length is adjustable from 0.25mm to 2mm according to the areas and purposes of the therapy; and needle tips are disposable, enabling the same handpiece with a fresh needle tip and guide to be used on multiple patients. The principal author of this study treated all of the research participants herself.

All participants received their professional scaling and dental hygiene instructions before any intervention. Local infiltration anesthesia was applied to anesthetize the area before the procedure began, followed by a Dermapen device. Gingival tissue thickness was assessed by starting 1.5 millimeters below the gum line. A number 15 endo file with an elastic stopper was placed vertically into the tissue underneath until it touched a firm floor, indicating bone. The length between the stopper and the file tip was calculated with a computerized measuring device (Venier caliper (Thermisto TH-M61, Bernstadt, Germany)) to assess tissue thickness [11]. The needle penetration level was modified to correspond with the previous gingiva thickness. The Dermapen was held at right angles to the surface and moved in horizontal, vertical, and diagonal directions. This procedure was done four to five times through the area until minor bleeding and redness were detected. These indications signaled that the micro-needling had achieved the desired penetration level to stimulate healing and reduce hyperpigmentation.

Next, a paste was made by mixing 1,000 mg/mL of ascorbic acid powder with ampule solution in a dapper dish. The paste was administered to the gingival mucosa for 10 minutes. After the treatment, no pack was placed in the therapeutic region. To reduce the risk of irritation to the gums, individuals were directed not to consume acidic or warm drinks for 1 day and not to brush the treated region for a single day. A prescription of Augmentin 625mg (BD) and Xerodol-sp (TD) was also provided for three days. Three case studies are included below to demonstrate the clinical outcomes of microneedling treatment.

## Results

Case 1

**Figure 2:**
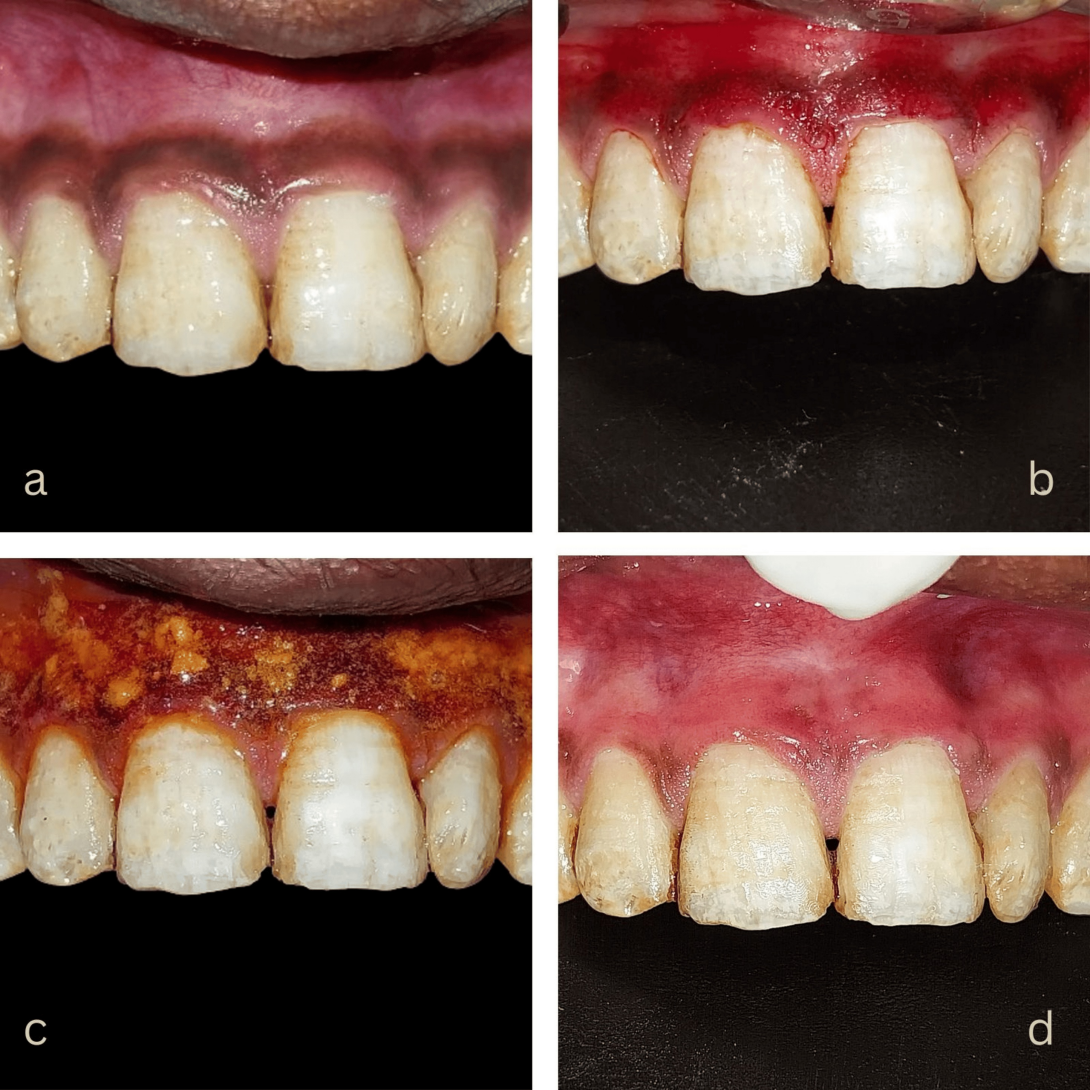
Microneedling treatment in Case 1: (a) Pre-operative photograph, (b) microneedling until mild erythema observed, (c) topical vitamin C application, and (d) healing at one month. Image Credit: Sana Mansuri.

A 27-year-old healthy male reported gingival discoloration in the upper anterior region. He was chewing tobacco for five years, consuming five to seven packets every day. The patient discontinued the habit a year before and wanted a depigmentation procedure to enhance the appearance of his smile. Therefore, we performed a microneedling procedure to address his gingival discoloration. Figures 2a-2d show depigmentation improvement at one month and three months post-procedure.

Case 2

**Figure 3:**
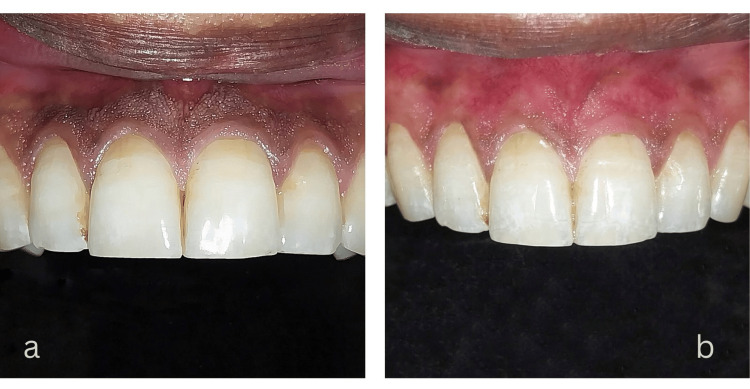
Microneedling outcome in Case 2: (a) Baseline pigmentation and (b) three months post-treatment. Image Credit: Sana Mansuri.

A 22-year-old male with a history of mild gingival hyperpigmentation dating back to adolescence, with no identifiable external factors such as smoking or systemic diseases. He had not used any medications that could influence pigmentation. The same Dermapen device was used with settings like Case 1. Following the microneedling treatment, his gingival color improved significantly over three months, with Figures 3a, 3b illustrating the progressive depigmentation.

Case 3

**Figure 4:**
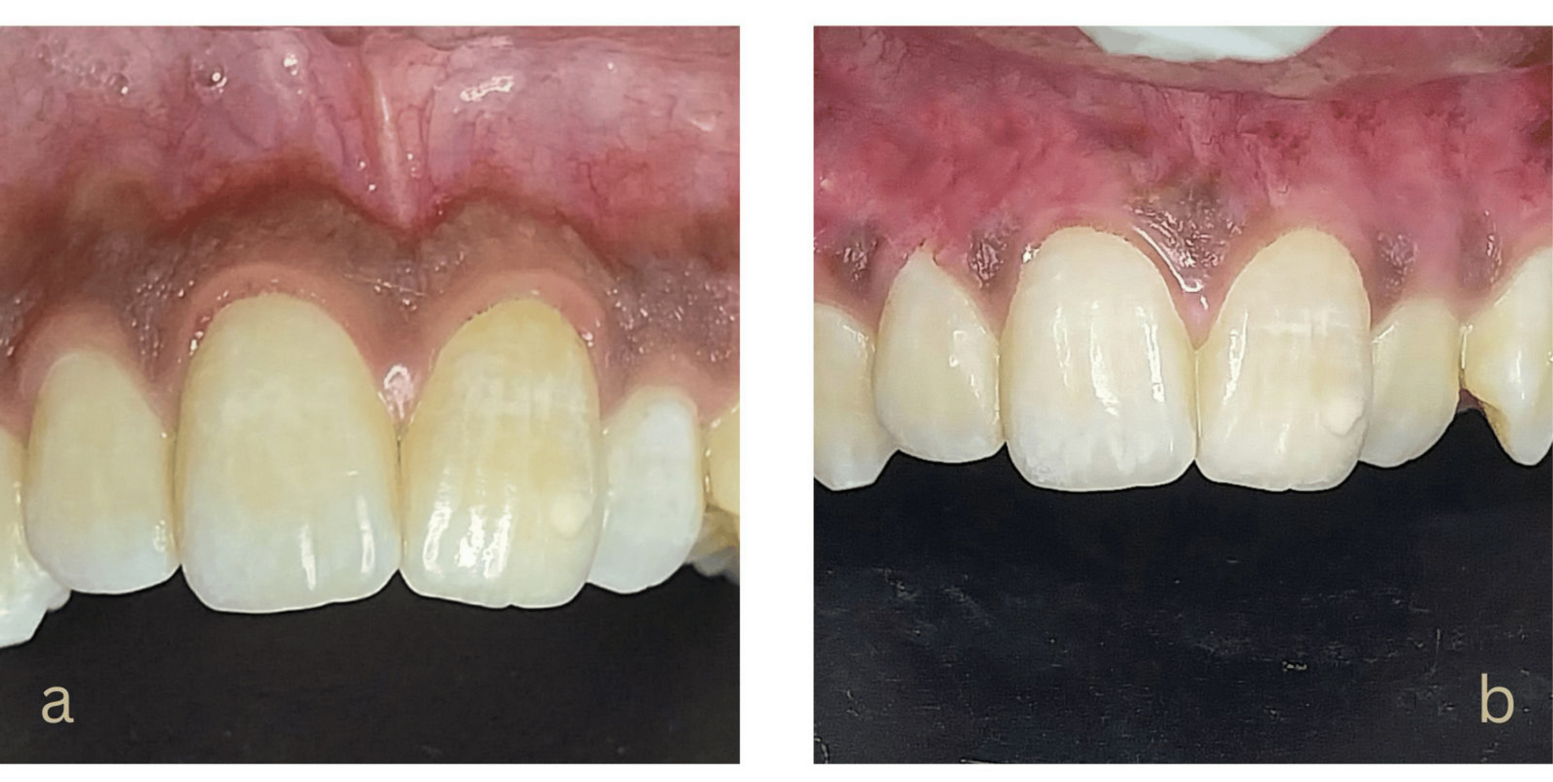
Clinical images from Case 3: (a) Pre-treatment pigmentation and (b) results after three months. Image Credit: Sana Mansuri.

A 15-year-old male presented with moderate physiological gingival hyperpigmentation. He did not have any previous record of smoking, systemic illnesses, or substances that could have influenced gingival color. His family and social histories were unremarkable. The patient underwent a microneedling procedure to address the pigmentation in settings like Case 1. However, due to his misaligned teeth and the deep contours of his gingiva, the Dermapen could not access certain regions. As a result, depigmentation was not achieved in these inaccessible areas. Despite these challenges, significant improvement was observed in the accessible regions at one and three months post-treatment (Figures 4a, 4b).

**Figure 5:**
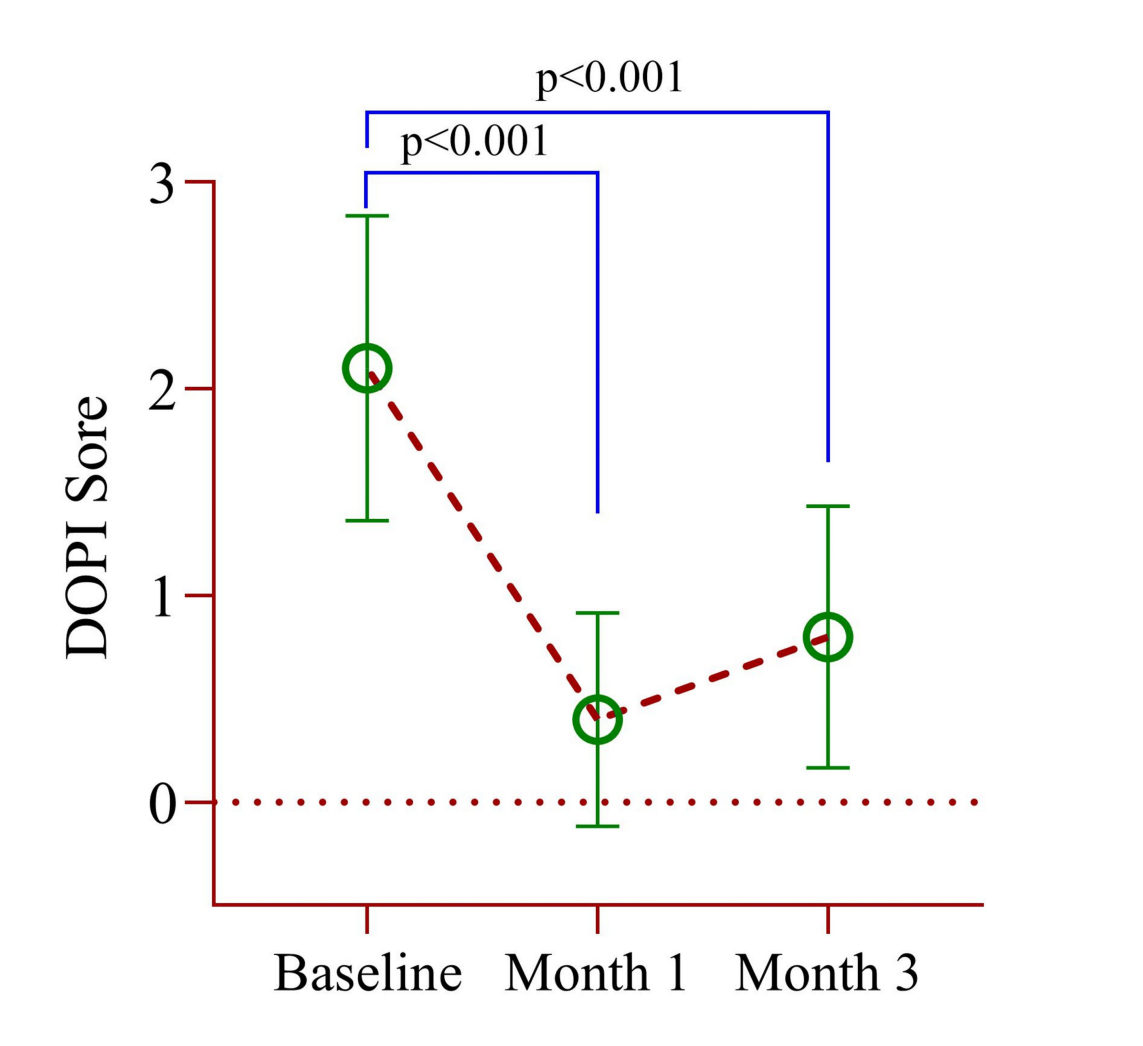
A mean difference of DOPI score between baseline, month 1, and month 3. Paired sample t-test was used to estimate the p-value. DOPI: Dummett oral pigmentation index Illustration Credit: Md Ahsanul Haq.

Ten participants took part in our study, with an average age of 23.00 ± 4.690 years. No participants withdrew from the study. The gender distribution was balanced, with equal representation of men (n=5) and women (n=5) (Table 1). The mean DOPI score at baseline was 2.10 ± 0.738, which decreased significantly (p<0.001) to 0.40 ± 0.516 after one month and slightly increased to 0.80 ± 0.632 after three months but was still considerably lower (p<0.001) than baseline (Table 2). Following therapy, DOPI scores significantly reduced, with a mean change of 1.3 ± 0.106 (95% CI: 1.572-0.348, p<0.001) (Figure 5).

**Table 1:** Gender representation among study subjects.

Gender	No. of participants (n)	Percentage (%)
Female	5	50
Male	5	50
Total	10	100

**Table 2:** Comparison of parameters at baseline, one month, and three months using Dummett oral pigmentation index (DOPI). SD: Standard deviation

Case no.	Baseline (B)	1 month (1M)	3 months (3M)	Age of participants
Case 1	2	0	0	27
Case 2	2	0	1	22
Case 3	3	1	1	15
Case 4	1	0	0	22
Case 5	2	1	0	25
Case 6	3	0	1	30
Case 7	2	0	1	26
Case 8	1	0	1	16
Case 9	3	1	2	21
Case 10	2	1	1	24
Mean	2.1	0.4	0.8	22.8
SD	0.737865	0.5163978	0.632456	4.685676

**Figure 6:**
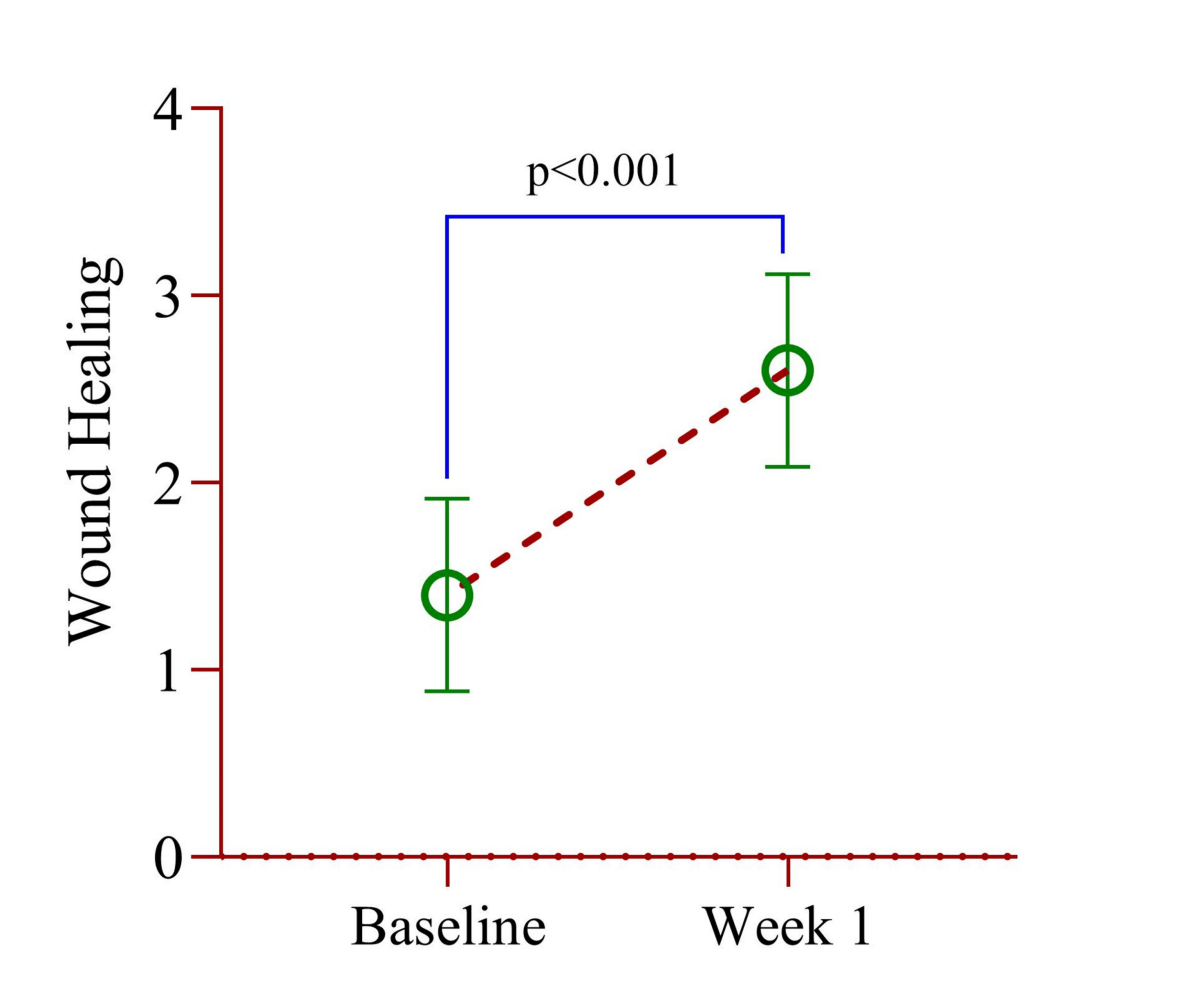
Mean difference of wound healing index between baseline and week 1. Paired sample t-test was used to estimate the p-value. Illustration Credit: Md Ahsanul Haq

Due to the limited number of participants, a nonparametric kernel-weighted local polynomial regression model was used to assess changes in the DOPI score from baseline to months 1 and 3. Compared to baseline, a significant decrease of 1.64 units at month 1 and 1.25 units at month 3 was observed (Table 3). There was no pain or tenderness reported. After two weeks, these symptoms had subsided, and the gingiva had returned to a pink appearance. To evaluate the invasiveness of the microneedling technique, wound healing scores were assessed at baseline and after one week. The mean wound healing score increased significantly from 1.40±0.52 at baseline to 2.60±0.52 after one week (p<0.001). Both scores had the same standard deviation, indicating similar group variability (Figure 6).

**Table 3:** Comparison of DOPI score between baseline to month 1 and month 3. Notes: A non-parametric kernel-weighted local polynomial regression model was used to estimate the p-value. A p-value of <0.05 was considered significant. DOPI: Dummett oral pigmentation index

Time of intervention	Estimate (95% CI)	P-value
Baseline	Ref. (0)	
Month 1	-1.64(-2.28, -1.12)	<0.001
Month 3	-1.25(-1.88, -0.72)	<0.001

The non-parametric polynomial regression model revealed a significant increase (p < 0.001) of 1.20 units in the wound healing index at week 1 compared to baseline (Table 4). Additionally, an effect size of 1.16 units was observed, indicating that wound healing within this short period was statistically and clinically meaningful. In summary, the paired t-test results show a significant improvement in wound healing from baseline to one week, with a large effect size indicating the magnitude of this improvement. The correlation analysis indicates a weak and non-significant relationship between wound healing scores at the two time points.

**Table 4:** Comparison of wound healing index between baseline to week 1. Notes: A non-parametric kernel-weighted local polynomial regression model was used to estimate the p-value. A p-value of <0.05 was considered significant.

Time of intervention	Estimate (95% CI)	P-value
Baseline	Ref. (0)	
1 week	1.20(0.71, 1.69)	<0.001

## Discussion

Oral hyperpigmentation is either physiological or pathological. Depending on the underlying condition, pathological hyperpigmentation can be categorized as exogenous or endogenous [12]. Exogenous pigment [4] can result from drugs, cigarette smoking, mercury tattoos, or metallic contact. Endogenous hyperpigmentation can be related to endocrine problems, disorders, prolonged discomfort, and reactive/neoplastic disorders [13]. Tyrosinase is a key enzyme in melanin biosynthesis, playing a crucial role in pigmentation by oxidizing tyrosine to produce the precursor dopaquinone [14]. The coloration of skin and mucosa involves four primary pigments: melanin, carotenoids, reduced hemoglobin, and oxygenated hemoglobin [15,16]. The principal pigment accounting for the gingival coloration is melanin, which is generated by melanin cells in the lowest layer of the gums. Each melanocyte can transfer melanosomes (melanin transporters) to approximately 30 to 40 keratinocytes [8].

**Figure 7:**
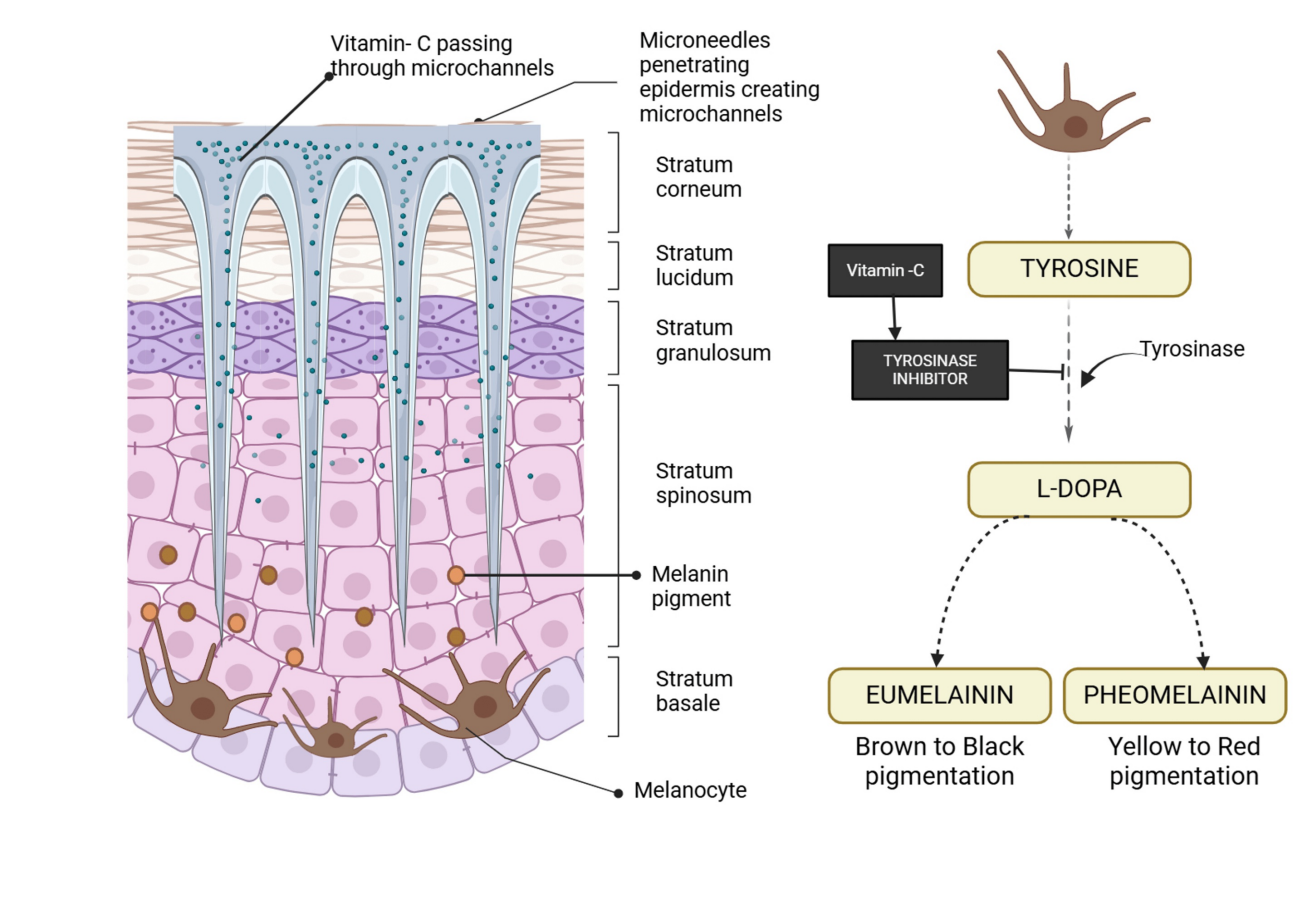
Mechanism of Dermapen and the effect of vitamin C on melanin. Notes: This figure was drawn using the premium version of BioRender [9] (https://biorender.com/), accessed on January 13, 2025, with the agreement license number AY27SUEQEL. Illustration Credit: Sana Mansuri.

Ascorbic acid (vitamin C) [13] inhibits the production of dopaquinone, thus reducing melanin synthesis. Also, it regulates the activity of tyrosinase and related enzymes (TRP-1 and TRP-2), disrupts melanosome distribution in keratinocytes, and interferes with pigmented keratinocyte development [17,18]. Our study employed a novel approach by combining microneedling with local application of ascorbic acid for gingival depigmentation. Microneedling is generally more affordable than laser treatments and advanced surgical techniques. Its versatility allows for use on various skin types and degrees of hyperpigmentation, and it can be performed with more straightforward tools like the Dermapen. However, it may be less effective for more extended periods. We used the Derma-pen Ultima A1, which features adjustable needle depths (0.25 to 2.5 mm) and a compact design for customized treatments [19]. The rapid, pulsating movement of the needles stimulates collagen production and enhances the absorption of therapeutic serums by creating microchannels in the skin [20]. Microneedling enhances blood flow and epidermis penetration by creating microconduits that allow therapeutic drugs to penetrate more efficiently (Figure 7) [4]. Microneedling-induced micro-injuries start a wound-healing pathway that produces development factors such as platelet-derived growth factors, transforming growth factors, connective tissue growth factors, and fibroblast growth factors. These responses promote collagen production and overall skin rejuvenation [21].

Our outcomes agree with earlier research that established the successful use of microneedling treatment to repair damaged skin and address pigmentation problems. For example, Dhanashree et al. found both Vitamin C mesotherapy and the scalpel technique effective for gingival depigmentation, with mesotherapy showing lower postoperative pain scores [22]. Another study by Mostafa et al. concluded that microneedling combined with topical ascorbic acid is an effective and minimally invasive method for gingival discoloration [4,23]. In Case 1, significant improvement was seen in the first month, with the DOPI score reducing from 2 to 0. This improvement was maintained at a score of 1, indicating that despite the pathological depigmentation, the microneedling procedure effectively provided substantial results. In Case 3, the DOPI score was reduced from 2 to 1. However, complete depigmentation was not achieved in regions with deep contours due to the Dermapen's inaccessibility. This limitation led to partial re-pigmentation in those areas. These findings suggest that while microneedling is generally effective, anatomical challenges can affect the outcome, highlighting the need for technique modifications or adjunctive treatments in some instances.

Apart from gingival depigmentation, Dermapen is used for various treatments. Al-Naggar et al. found microneedling with bleomycin more effective and less painful for plantar warts than bleomycin injection alone [24]. Ozsagir et al. found that micro-needling with i-PRF improved gingival thickness in patients with thin gingiva [17]. Mostafa et al. found that using coconut and sesame oils reduced gingival inflammation with micro-needling [25]. Zdunska et al. highlighted microneedling's benefits for skin regeneration, collagen production, and treating conditions like photoaging and scars [20]. Alster and Graham reviewed micro-needling's efficacy in dermatology for rejuvenation and acne scars [26]. Our findings support the microneedling procedure as a viable treatment for gingival hyperpigmentation, providing a barely invasive option with rapid recovery. However, anatomical challenges, such as deep gingival contours and misaligned teeth, can affect the outcome.

Limitations of this study

Case 1, with a 27-year-old male, produced remarkable results but underlined the importance of precise control to avoid over-depigmentation. Case 2, with a 22-year-old, demonstrated lesser results in areas near the interdental and marginal regions. In Case 3, with a 15-year-old with irregular gingival contours, the approach labored in difficult-to-reach places, emphasizing anatomical limits.

Futuristic research approach

We expect to conduct a well-designed prospective multicenter study to generalize our data throughout the country.

## Conclusions

Microneedling in adjunct with topical vitamin C is a successful and safe treatment for gingival tissue depigmentation, with most participants reporting positive outcomes. While the technique shows significant promise, further studies are necessary to refine the approach and address specific anatomical challenges. Overall, microneedling offers a viable, minimally invasive treatment option for patients seeking aesthetic improvements in gingival pigmentation.
